# Hepatic Echinococcosis as an Incidental Finding in a Patient With a Perforated Gastric Ulcer: A Case Report

**DOI:** 10.7759/cureus.104438

**Published:** 2026-02-28

**Authors:** Ricardo José Razera, Joana Loury Pinheiro de Oliveira, Maria Carolina Brandão Moran, Mariana S Kajita, Yuri dos Santos Buscariolli, Isabella Lazaretti Morato Castro, Marcos Vinicius da Silva, Jose C Ardengh

**Affiliations:** 1 Infectious Diseases, Emílio Ribas Institute of Infectious Diseases, São Paulo, BRA; 2 General Surgery, Hospital Municipal Antônio Giglio, Osasco, BRA; 3 General Surgery, Hospital Municipal Antônio Gilglio, Osasco, BRA; 4 General Surgery, Complexo Hospitalar Heliópolis, São Paulo, BRA; 5 Family and Community Medicine, Hospital de Força Aérea de São Paulo, São Paulo, BRA; 6 Gastrointestinal Endoscopy Department, Hospital das Clínicas de Ribeirão Preto, Ribeirão Preto, BRA; 7 Diagnostic Imaging, Universidade Federal de São Paulo, São Paulo, BRA; 8 Digestive Endoscopy, Hospital Moriah, São Paulo, BRA

**Keywords:** acute surgical abdomen, cystic echinococcosis (ce), hepatic echinococcosis, neglected disease, stomach ulcers

## Abstract

Echinococcosis is a zoonotic helminth infection with global distribution and a large public health impact. In Brazil, cases are traditionally associated with the Southern region, but mortality records show a substantial proportion of cases occurring in the Northeast, supporting underrecognition outside historically endemic areas.

Herein, we report a case of a 67-year-old man who presented in early April 2025 with an acute abdomen due to a perforated gastric ulcer. He reported childhood exposure to sheep and dogs in rural Paraíba. Computed tomography (CT) demonstrated pneumoperitoneum and an incidental calcified hepatic cystic lesion in segment VII. Emergency laparotomy confirmed the perforated gastric ulcer, and the defect was repaired with ulcerorrhaphy and an omental patch. The hepatic lesion was excised en bloc with an intact cyst wall. The cyst was not opened or aspirated, and no spillage was observed. Histopathology demonstrated features consistent with cystic echinococcosis (CE). Albendazole was initiated and titrated to a maintained regimen of 400 mg orally twice daily. Serology was requested, and results were not available at the time of writing this report. Follow-up CT showed complete resection with no abdominal collections or evidence of dissemination. Lower limb weakness after intensive care was observed and attributed to severe deconditioning and critical illness-related neuromuscular weakness.

This case highlights that CE can be encountered unexpectedly during emergency surgery in non-endemic urban settings and underscores the importance of safe intraoperative handling, clear operative documentation, and structured follow-up.

## Introduction

Cystic echinococcosis (CE), also referred to as hydatid disease, is caused by the larval stage of* Echinococcus granulosus* *sensu lato*. Humans acquire infection by ingesting parasite eggs shed in the feces of definitive hosts, most commonly dogs. After ingestion, embryos reach the liver via the portal circulation and develop into slowly growing cysts that may remain asymptomatic for years [1]. CE is strongly linked to pastoral and agricultural environments and remains a neglected zoonotic disease with substantial health and economic consequences [2].

Pan American Health Organization (PAHO) and World Health Organization (WHO) summaries indicate that more than one million people are affected by echinococcosis at any given time. WHO estimates for 2015 further attribute approximately 19,300 deaths and about 871,000 disability-adjusted life years per year to echinococcosis worldwide. The frequently cited estimate of an annual global economic burden of approximately three billion US dollars is derived from global burden analyses published in 2006 that combined human treatment costs with livestock production losses and accounted for underreporting [3].

In Brazil, CE is most frequently reported in the Southern region, where sheep husbandry is common. However, national surveillance and mortality analyses indicate that cases and deaths occur across multiple regions, including the Northeast [4,5]. These patterns support the need to consider CE in the differential diagnosis of hepatic cysts, even in large urban centers that receive migrants from rural areas. Most infections are acquired through close contact with infected rural dogs and exposure to contaminated soil, food, or water [6,7].

We describe a case of incidental hepatic CE discovered during emergency surgery for a perforated gastric ulcer in a patient from the Brazilian Northeast region. The report focuses on intraoperative decision-making and postoperative management when CE is not suspected preoperatively.

## Case presentation

A 67-year-old man, originally from a rural municipality in Paraíba in the semiarid Northeast Brazil, was evaluated in July 2025 at the Neglected Tropical Diseases outpatient clinic of a tertiary infectious diseases hospital in São Paulo after discharge from a prolonged hospitalization. He had lived in metropolitan São Paulo since adolescence. During childhood, he raised sheep and chickens, used well water, and had close contact with dogs, which are relevant exposure factors for CE.

**Figure 1:**
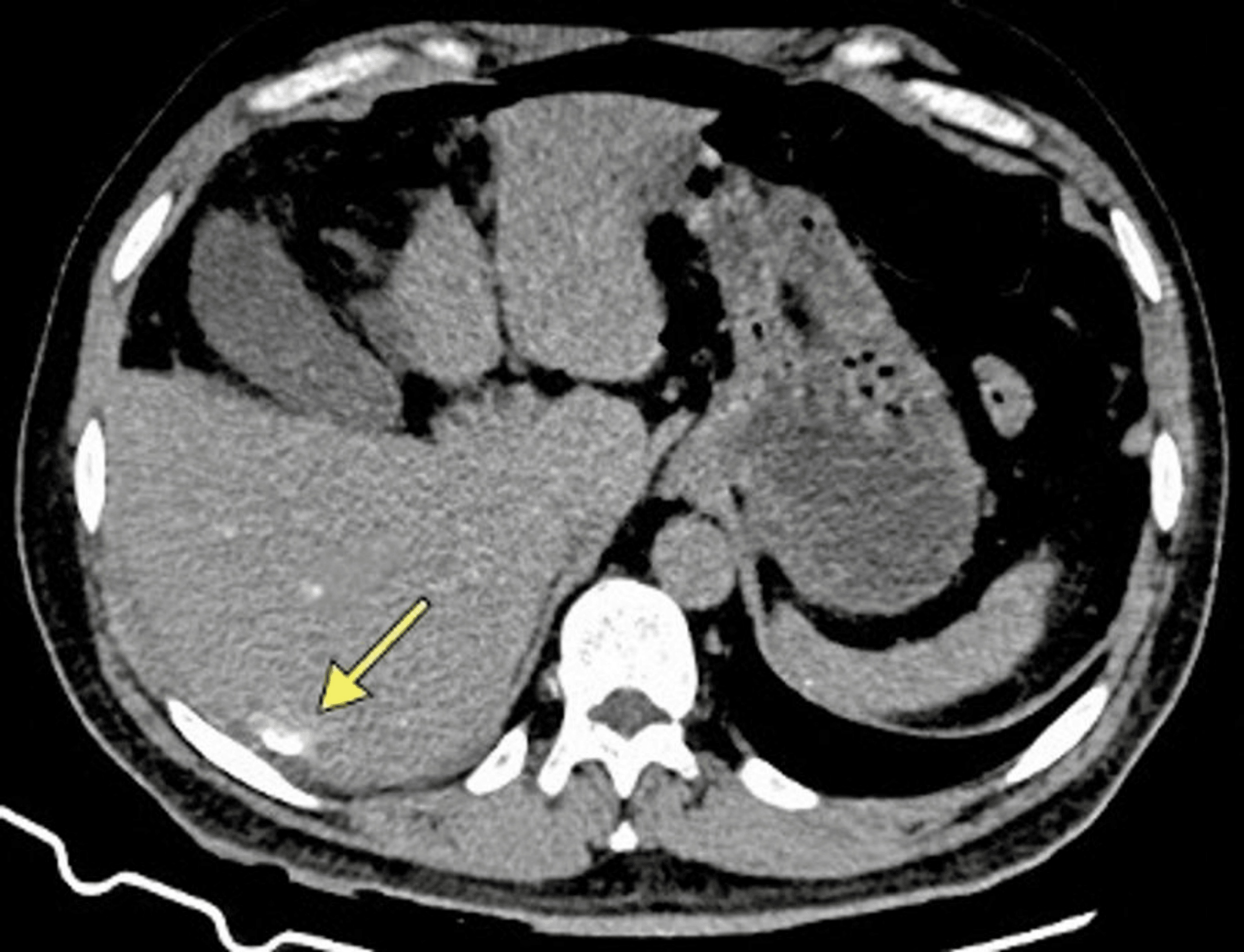
Preoperative contrast-enhanced abdominal CT Axial CT image obtained at emergency admission. The image demonstrates free intraperitoneal air compatible with a perforated hollow viscus. An incidental calcified cystic lesion is visible in hepatic segment VII (arrow), measuring 5.3 cm in maximal diameter, with peripheral rim calcification. Because the WHO-IWGE cyst classification is ultrasound based, any stage inference from CT alone should be interpreted cautiously, and formal ultrasound staging was not performed in this case. CT, computed tomography; WHO-IWGE, World Health Organization Informal Working Group on Echinococcosis.

He reported chronic epigastric pain and intermittent dyspepsia lasting approximately six years, with progressive worsening in early 2025, describing poor oral intake and weight loss. He denied fever, vomiting, or diarrhea before the acute event. On April 7, 2025, he presented to the emergency department with severe abdominal pain, nausea, syncope, and signs of shock. On arrival, he was hypotensive (80/50 mmHg), tachycardic (114 beats per minute), tachypneic (26 breaths per minute), and afebrile (36.5°C). Physical examination revealed diffuse abdominal tenderness and peritoneal signs. Admission laboratory values showed hemoglobin at 18.0 g/dL, leukocyte count of 6,440 cells/mm³, platelet count of 273,000 cells/mm³, C-reactive protein level at 128 mg/L, sodium 134 mEq/L, potassium 4.4 mEq/L, urea 42 mg/dL, creatinine 1.15 mg/dL, total bilirubin 1.98 mg/dL (direct 0.28 mg/dL), amylase 66.6 U/L, prothrombin time 14.0 seconds with INR 1.24, and activated partial thromboplastin time 27.1 seconds. Contrast-enhanced abdominal computed tomography (CT) showed free intraperitoneal air consistent with a perforated hollow viscus and a 5.3 cm calcified cystic lesion in hepatic segment VII (Figure 1). The patient underwent emergent exploratory laparotomy.

The patient underwent emergency exploratory laparotomy with ulcerorrhaphy and an omental patch for the perforated gastric ulcer. During the operation, a firm subcapsular hepatic lesion was identified in segment VII and was excised. The cyst was removed en bloc with preservation of the cyst wall. It was not opened, ruptured, aspirated, or irrigated, and no macroscopic spillage was observed. No additional field isolation with scolicidal agents was used because echinococcosis was not suspected at the time. His postoperative course required a prolonged intensive care unit stay totaling 57 days, complicated by multiple healthcare-associated infections, grade 3 sacral pressure injury, and severe deconditioning. Detailed ICU microbiology, ventilator days, and specific complication timelines were not available in the records reviewed for this case report, and this limitation is explicitly acknowledged.

**Figure 2:**
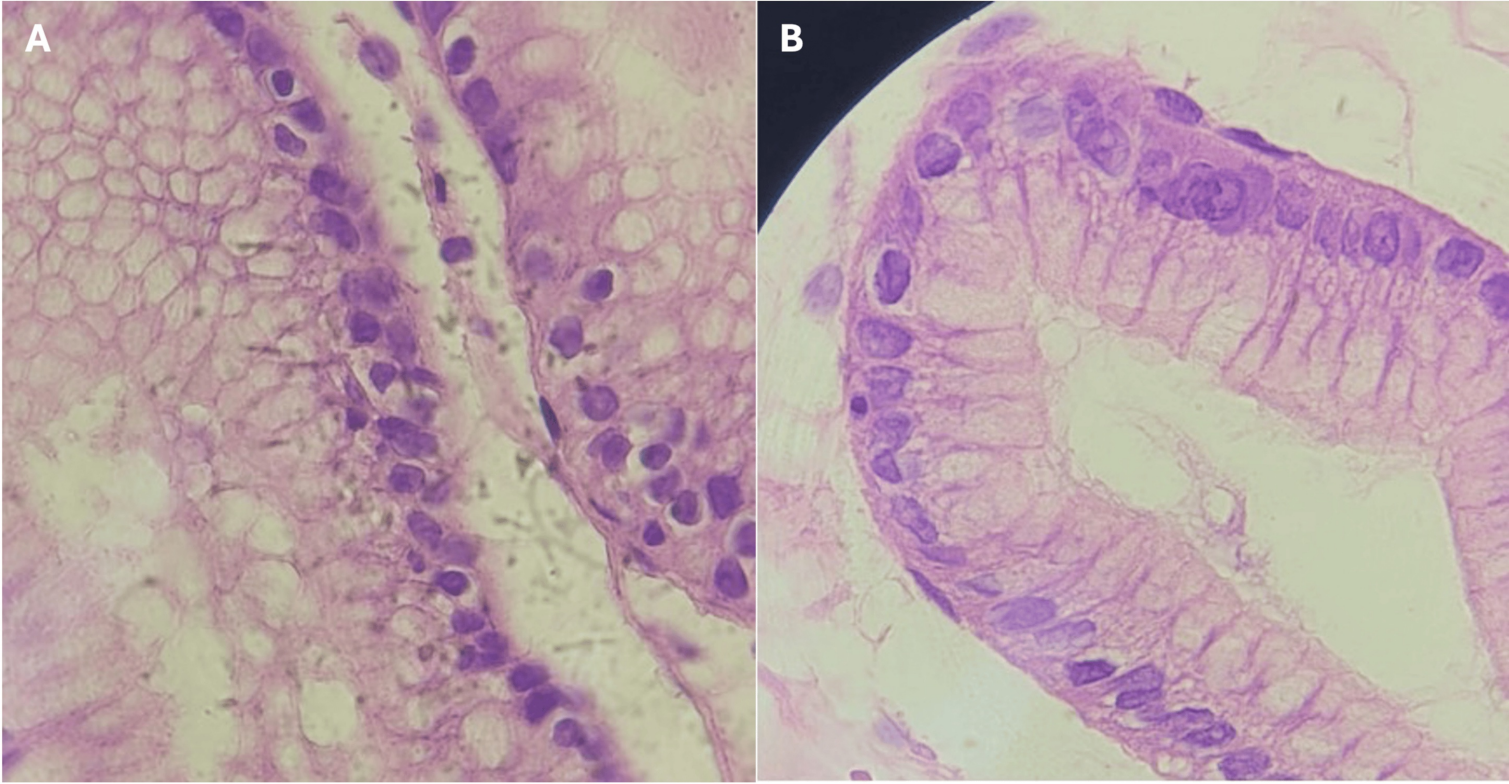
(A) Laminated membrane of the echinococcal cyst wall, with histopathological detail of the hydatid cyst wall. The image demonstrates the characteristic acellular laminated membrane with typical eosinophilic stratified appearance, a pathognomonic finding in cystic echinococcosis caused by Echinococcus granulosus sensu lato. This PAS-positive hyaline membrane represents the outer protective layer of the parasite cyst (H&E stain, original magnification 400x). (B) Brood capsule of the hepatic hydatid cyst, with histopathological examination of the hepatic cyst. Microscopy revealed a brood capsule (proliger capsule) with characteristic layered cellular architecture, consistent with Echinococcus species. The presence of this structure confirmed the parasitic nature of the lesion and supported the diagnosis of cystic echinococcosis (H&E stain, original magnification 400x).

Pathological examination of the hepatic lesion demonstrated an acellular laminated membrane and parasitic structures consistent with cystic echinococcosis (Figures 2A-2B). Protoscolices or hooklets were not described in the available pathology report, and there was no description of biliary communication. Gastric ulcer biopsy demonstrated chronic active gastritis with ulceration and was negative for *Helicobacter pylori *and malignancy.

Following histopathological confirmation, albendazole therapy was initiated and titrated to a maintained regimen of 400 mg orally twice daily, taken with fatty meals to improve absorption [8,9]. A three-month course was planned as adjuvant therapy because the cyst had been manipulated during emergency surgery, and the preoperative stage was uncertain [1,9]. Body weight was documented as 69 kg, corresponding to approximately 11.6 mg/kg/day for the maintained regimen of albendazole 400 mg orally twice daily. Baseline and periodic monitoring of liver transaminases and complete blood count was planned during therapy in line with guideline recommendations [1,9]. Serology for echinococcosis was requested, and results were not available at the time of manuscript preparation. Ultrasonography was not performed, and staging therefore remains speculative.

Follow-up contrast-enhanced CT performed during hospitalization demonstrated complete resection of the hepatic lesion with no abdominal collections and no radiological evidence of intraperitoneal dissemination. The patient reported lower limb weakness after discharge. This was attributed clinically to severe deconditioning and suspected critical illness-related neuromuscular weakness after prolonged intensive care.

## Discussion

This case describes a case of incidental hepatic CE encountered during emergency surgery for a perforated gastric ulcer. Although the liver is the most common site of CE, diagnosis during laparotomy performed for unrelated pathology is uncommon [10]. When CE is unexpectedly encountered intraoperatively, many authors recommend completing the primary emergency procedure and deferring definitive CE management when feasible, especially when the cyst appears inactive, and there is no complication requiring urgent intervention [11]. In the present case, excision occurred during the index emergency laparotomy because the lesion was subcapsular, technically accessible, and could be removed en bloc without opening the cyst. In an ideal setting, an alternative approach would have been to document the lesion, avoid additional manipulation, complete the ulcer repair, and arrange staged evaluation by a hepatobiliary team with ultrasound-based classification and multidisciplinary planning. We explicitly acknowledge that both strategies are reasonable depending on the operative context, available expertise, and intraoperative risk assessment.

The WHO Informal Working Group on Echinococcosis classification is ultrasound based and guides management according to cyst activity [1]. In this case, only computed tomography was available and appropriate to the clinical scenario in an urgent care setting, showing peripheral calcification and internal septations, which may suggest an inactive or transitional cyst. Because CT cannot reliably replace ultrasound for staging, we report this interpretation as an inference with clear limitations, and formal WHO staging was not performed.

In an ideal setting, preoperative recognition of possible CE would allow risk mitigation and shared decision-making. If the primary indication is an emergency for perforation, the safest approach is often to avoid unnecessary cyst manipulation, protect the operative field if CE is suspected, and plan elective management after stabilization. When excision is performed unexpectedly, documentation of whether the cyst was opened or spilled is essential because it directly informs the risk of anaphylaxis, secondary peritoneal disease, and the need for adjuvant therapy and follow-up.

The inadvertent manipulation and excision of a hydatid cyst without prior recognition can expose the surgical team and patient to preventable hazards, including anaphylaxis and secondary peritoneal echinococcosis [12-15]. In this case, the cyst was removed intact without gross spillage, and no immediate allergic reaction occurred. Nevertheless, the absence of preoperative suspicion meant that additional protective measures were not used, which emphasizes the importance of maintaining CE in the differential diagnosis of hepatic cysts even in non-endemic urban settings.

When CE is suspected or encountered intraoperatively, recommended precautions focus on minimizing rupture and contamination, protecting the operative field, and being prepared to treat allergic reactions promptly [1,8,16]. Choice and use of scolicidal agents and other intraoperative measures should follow institutional protocols and specialist guidance, especially when cystobiliary communication is possible. In this event, these additional precautions were not implemented because CE was not suspected before or during the initial approach to the emergency surgery. If the lesion is retrospectively interpreted as an inactive or transitional cyst based on CT features, observation would often be acceptable in non-emergency contexts. In this emergency setting, the decision to excise was driven by intraoperative accessibility and the ability to remove the cyst en bloc without rupture. We therefore frame the management as a pragmatic intraoperative decision rather than as a recommendation to excise all calcified cysts, and we emphasize that ultrasound-based staging and elective planning remain preferred when circumstances allow.

Also, the differential diagnosis of cystic liver lesions includes simple hepatic cysts, cystadenomas, pyogenic or amebic abscesses, neoplasms with cystic degeneration, and metastatic disease [17,18]. Intraoperative differentiation can be challenging, particularly when the cyst has an atypical appearance such as multiloculation, turbid contents, or thick walls. The American College of Gastroenterology (ACG) guidelines on management of focal liver lesions recommend that echinococcosis be considered in all patients with complex hepatic cysts, particularly those with a history of rural origin, contact with dogs, or exposure to sheep farming, even in countries without traditional endemicity [17]. In this case, histopathological examination was essential for definitive diagnosis, demonstrating the characteristic acellular laminated membrane pathognomonic of echinococcal infection.

This case also supports the need to recognize that CE occurs beyond the historically endemic areas of Southern Brazil. Published reviews and surveillance reports have documented Echinococcus species and the associated human disease across multiple Brazilian regions, including the Northeast [4-7]. The patient reported childhood exposure to sheep, dogs, and untreated water in rural Paraíba, which is compatible with CE acquisition despite later residing in an urban center. Mortality data showing a high proportion of deaths in the Northeast further suggest delayed recognition and underdiagnosis in that region [5].

Guidelines generally recommend albendazole as first-line medical therapy for CE, often using a fixed adult regimen of 400 mg twice daily or a weight-based dose of 10-15 mg/kg/day divided twice daily [8,9]. For postsurgical or manipulated cysts, adjuvant therapy is commonly recommended for at least three months, with longer courses considered when spillage occurs or when viable elements are suspected [1,9]. Monitoring typically includes baseline and periodic liver function tests and complete blood count during therapy, with the frequency individualized to patient risk and local protocols [1,9]. Under the cited circumstances, intact removal without spillage supported a finite adjuvant course, while the absence of ultrasound staging and limited pathology descriptors warranted a cautious follow-up plan.

This report has several limitations. Formal ultrasound-based staging was not performed, and retrospective staging from computed tomography alone is uncertain. Intensive care unit microbiology data were not available for inclusion, which limits objective characterization of the acute perforation severity and the drivers of the prolonged intensive care course. Serology was requested, but results were not available at the time of writing, and molecular genotyping was not performed. Long-term follow-up outcomes are also pending. Current guidelines suggest that postoperative imaging follow-up may be considered at approximately 3, 6, and 12 months, and then periodically thereafter based on cyst characteristics and clinical context [1,9].

The most important learning points are as follows: CE should be considered in the differential diagnosis of hepatic cysts; even in non-endemic urban settings, a thorough epidemiological history is essential for recognizing CE risk in patients presenting outside endemic areas; and if CE is suspected during surgery, operative field protection and avoidance of cyst rupture are key safety priorities. When a cyst is excised unexpectedly, documentation of cyst integrity and any spillage is critical for risk assessment and follow-up planning. Histopathological examination can establish the diagnosis when serology or ultrasound staging is not available, and follow-up imaging and clinical reassessment are important; recommendations should be individualized when long-term outcomes are not yet known.

## Conclusions

This report describes incidental hepatic cystic echinococcosis identified during emergency surgery for a perforated gastric ulcer in a patient from rural Northeast Brazil who later presented for follow-up in São Paulo. The case emphasizes that CE can be encountered unexpectedly in non-endemic settings and that intraoperative recognition, safe handling, and clear documentation of cyst integrity are central to minimizing avoidable complications. Because serology and long-term follow-up results were not yet available, conclusions about optimal staging and duration of therapy should be interpreted cautiously.
